# Is hate speech detection the solution the world wants?

**DOI:** 10.1073/pnas.2209384120

**Published:** 2023-02-27

**Authors:** Sara Parker, Derek Ruths

**Affiliations:** ^a^School of Computer Science, McGill University, Montreal, QC H3A 0E9, Canada

**Keywords:** hate speech, natural language processing, machine learning

## Abstract

The machine learning (ML) research community has landed on automated hate speech detection as the vital tool in the mitigation of bad behavior online. However, it is not clear that this is a widely supported view outside of the ML world. Such a disconnect can have implications for whether automated detection tools are accepted or adopted. Here we lend insight into how other key stakeholders understand the challenge of addressing hate speech and the role automated detection plays in solving it. To do so, we develop and apply a structured approach to dissecting the discourses used by online platform companies, governments, and not-for-profit organizations when discussing hate speech. We find that, where hate speech mitigation is concerned, there is a profound disconnect between the computer science research community and other stakeholder groups—which puts progress on this important problem at serious risk. We identify urgent steps that need to be taken to incorporate computational researchers into a single, coherent, multistakeholder community that is working towards civil discourse online.

Hate speech is bad. Although its exact definition is debatable, the majority of people can agree that the spread of hate speech should be prevented as much as possible (1). Unfortunately, this has proven to be a difficult task on the Internet: online hate speech appears ubiquitous and is shaping more and more conversations on social media (2). Many stakeholders are trying to do something about this—among them, computer scientists, who have been eagerly building automated hate speech detection systems for multiple years (3). However, this major effort by the computer science (CS) community may be better directed toward building holistic solutions for online hate speech, rather than methodological problem-solving.

## Hate Speech Detection Is Hot in the CS Community

Whether a researcher trying to examine hate speech dynamics or a platform trying to prevent its spread, finding hate speech is an important first step to addressing it. Automated detection is therefore appealing for its ability to find hate speech much quicker and in vastly greater quantities than humans (4, 5). Automated methods also relieve the emotional burden of reading hundreds or potentially thousands of samples of hateful content from content moderators and users, who would otherwise be solely responsible for detecting and reporting hate speech.

However, automated hate speech detection is complicated—this complexity is what makes it such an interesting computer science challenge (6). Keywords that appear in hate speech lexicons may be used in ways that do not count as hate speech (e.g., the black community’s reclamation of the n-word) or words that are used as hate speech may only be hateful in a specific context or forum (e.g., “tr*nny” is a slur used to refer to members of the transgender community but is used to refer to a car’s transmission in auto enthusiast communities) (7). Additionally, hate speech often does not include keywords, but is distinguished by the overall impact of the content—somebody can attack someone without using a slur, or even derogatory vocabulary (e.g., referencing gas chambers in response to a Jewish person is undoubtedly anti-Semitic, but contains no words that are vulgar on their own) (8). Finally, the nature of hate speech changes: The means of cyberbullying evolve over time and across online communities, necessitating automated detection systems that can evolve with the online dynamics they track (9). As a result, online hate speech has become the subject of substantial interest in the computer science community, inspiring groundbreaking research in machine learning (ML) that leverages deep learning and unsupervised methods to detect hate speech in ways and on scales unattainable by humans.

As active researchers in this area for the past decade, it has been our experience that automated detection methods receive disproportionate attention from the computer science community—a trend that, we admit, we have been a part of as well. A quantitative study of the literature confirms this trend (see *SI Appendix*, *Appendix A* for details). When we search top computer science databases—the Association for the Advancement of Artificial Intelligence’s AITopics database or the Association of Computing Machinery’s Digital Library—for computational work on hate speech or offensive language, we find that almost 90% of papers were exclusively about automated detection. If we consider papers on hate speech appearing in top computer science conferences, we similarly find that 89% of these focus exclusively on demonstrating or testing methods for detecting hate speech.

## Detection Is Not a Solution

However, detection is just one small component of the much larger challenge of online hate speech. In fact, automated detection, despite its myriad benefits, is an extraordinarily messy response due to the complex logistical, financial, ethical, and technical questions that must be addressed by actual hate speech solutions. If a social media platform has the technical capacity to automatically detect and flag hate speech (and this is a big “if”!), they must still reckon with the choice of what to do when hate speech is detected. Is it automatically removed (thereby assuming the system is and always will be 100% accurate) or is it subject to review by a human moderator (thus requiring the platform to hire, train, and continuously pay a team of moderators) (10)? The platform would also need to consider the degree of involvement users have: for example, if users are still responsible for reporting harmful content, or if they can appeal decisions made by the system. Additionally, what constitutes “hate speech” may change over time; those responsible for creating and managing the automated detection system would need to create mechanisms to adapt to the ever-evolving nature of online hate speech and cyber-bullying (11). The reliability of the system would also need to be consistently tested to ensure that it remains accurate, given potential changes to the definition of hate speech. Finally, there are a variety of ethical and legal aspects of implementing automated detection that lack consensus. Is there reasonable justification for the monitoring of online content, given the proportionate harm of hate speech? Is removing hate speech a violation of the right of expression (12)? Does actively searching for hate speech make platforms responsible for its propagation? Do the answers to these questions change depending on the legal codes across countries (13)?

Given how complicated these questions are, the puzzle of automated detection is not the only challenge facing those hoping to address the spread of online hate speech. Finding hate speech, evidently, is almost as complex as responding to it.

## Making Sense of the Mess

Other stakeholders—specifically governments, nonprofits, and tech companies—reflect this complexity inherent to the hate speech problem, as evidenced by the wide diversity of solutions they are considering, discussing, and experimenting with. For instance, many social media platforms rely heavily on user moderation, while some advocacy organizations have begun coordinating counterspeech campaigns to directly respond to hate speech and dismantle the thinking that allows for it to occur (14). However, these isolated initiatives are evidently insufficient in stopping hate speech given the continued creation and proliferation of hate speech online, so the demand for a multi-stakeholder, holistic solution remains high (15). Although stakeholders other than computer scientists clearly recognize that hate speech is a layered issue, they do not necessarily agree on what to do about it.

To better understand how other essential actors are thinking about online hate speech, we analyzed the discourse from three key stakeholders who can really influence the responses to online hate speech: governments, nonprofits, and platforms (16) (See *SI Appendix*, *Appendix B* for details on our sources and methods). Despite the high volume of content about online hate speech produced by these stakeholders, we were surprised to find relatively few texts that were concerned with the detection of or specific responses to hate speech, rather than simply asserting it as a problem. Our subsequent relatively small sample (35 unique texts) included all English-language texts that discuss, reference, or feature hate speech response from government sources (like official national legislation, party platforms, and government-commissioned reports), nonprofits (statements on specific events, policy proposals), and major social networking platforms (official policies, news releases, articles, and blog posts) (See SI Appendix, Figure 1).

Each text contains the author-stakeholder’s stance on one or more aspects of an effort to mitigate online hate speech: who creates it, who is making it harmful and how, who is harmed, who has the responsibility to do something about it, and/or who decides what that responsibility is. Recall that our primary objective was to determine who a text held accountable for fixing the presence of online hate speech and how they should do it—with the idea that this is where automated hate speech detection would (or would not) be highlighted. In order to serve and give context to this narrow goal, we undertook a thorough deconstruction of each text’s worldview around each of the aspects mentioned above. We read each text with the goal of identifying what aspects of online hate speech the stakeholder was addressing, and then read them again to pinpoint who the stakeholder believed played a part in it. We would have gone further to also consider what the stakeholder believed should be done for a given aspect (i.e., use automated detection to do something about hate speech), but as we will see, views of stakeholders were so disengaged from automated detection that this part of our analysis was not necessary. Our systematic analysis of the texts allowed us to develop a framework for understanding text and stakeholder positions, consisting of “characters” (Individuals, Nonprofits, Platforms, and Governments) who play “roles” (Victim, Villain, Detector, Responder, and Governor) in the textual narratives about how to respond to online hate speech (Table 1). This framework gives us a way to systematically understand how stakeholders think about online hate speech: for instance, although Reddit does not explicitly state in their content policy that they believe they are solely responsible for defining what constitutes hate speech and what to do with users who engage in it (the role of the Governor), the lack of mention of other stakeholders (besides Individuals, as Victims, Villains, and sometimes Detectors) in the policy is indicative of who the platform believes should have the final say in hate speech solutions (i.e., platforms themselves) (19). When the framework is applied to all the texts in the sample, we readily identify trends in how different stakeholders think about the problem of online hate speech and its mitigation (Table 2).

**Table 1.** Characters and roles in hate speech response discourse

	Text 1: Liberal Party of Canada: Protecting Canadians from Online Harm (17)	Text 2: Tumblr’s Community Guidelines (18)
Villain	Platforms, as hosts of hateful content, are perpetrating harm.	Individuals, by creating hateful content, are perpetrating harm.
Victim	Individuals, who encounter hate speech online, are harmed.	No mention of anyone being targeted or affected by hate speech.
Detector	No mention of how hate speech is detected or found.	Individuals encounter hate speech on the platform and report it.
Responder	No mention of what response should be taken against online hate speech or by whom.	The Platform is responsible for processing complaints and removing content or users accordingly.
Governor	The Government is responsible for creating and enforcing rules pertaining to hate speech.	The Platform is responsible for creating and enforcing rules pertaining to hate speech.

In texts, we observed consistent narrative structures that involved entities doing certain things (e.g., creating hate speech, getting hurt by hate speech, finding hate speech). We call these the “roles” in the discourse. Characters were the entities that could be assigned to a given role. This table gives a sense for how the narratives from two texts might differ.

**Table 2.** The character–role framework as applied to several texts

	Character					
Text	Villain	Victim	Detector	Responder	Governor	Stakeholder
OHCHR: Report on online hate speech	Government			Platform	Government	G
HRW: Germany flawed social media law	Government	Individual				N
TikTok: Community guidelines	Individual			Platform	Platform	P

This is an example of how the Character–Role framework is applied to the texts to identify trends across stakeholder groups. See *SI Appendix*, *Appendix B* for the breakdown of these categories and the completed table with all texts.

We identified several key trends:

- Governments: government discourse emphasizes the need to respond to online hate speech and governing that response, but with little attention given to the actual act of detecting online hate speech. Governments do not think of themselves as responders, instead delegating that role to platforms. Furthermore, they do not assume that responding to hateful content involves removing it; rather, half of them assert that platforms should simply start by monitoring their content. That being said, governments do want to maintain control over hate speech solutions, either by creating and enforcing rules, receiving reports on hate speech detection from platforms, “understanding” hate speech, or collaborating with other actors on solutions.
- Nonprofits: discourse from nonprofits overwhelmingly focuses on the Villain–Victim relationship, with individuals being subject to harm as a result of online hate speech. Interestingly, the Villain is not always the perpetrator of hate speech: the majority of Villains are governments or platforms, who violate individual freedoms through content surveillance or censorship.
- Platforms: mainly discuss how they combat hate speech on their sites, sometimes mentioning automated detection but mostly shying away from the specific technical mechanisms. They primarily explain their user moderation policies: how users can report potentially harmful content, how that content is evaluated, and what happens if content is against their community guidelines. Platforms almost always assert themselves as predominantly responsible for maintaining safe online communities and mitigating online hate speech, seldom mentioning other characters beyond Individuals.

Although there is widespread agreement that the focus should be on protecting individuals (either as citizens or users) and that platforms are principally responsible for responding to hate speech, two key contradictions stood out to us.

1) Platforms are the only stakeholder that appear significantly concerned with detecting hate speech. Even though they sometimes mention automated detection, platforms do not appear inclined towards solely (or even primarily) relying on it, as many rely on users to report harmful content (every platform included in our sample has a mechanism allowing users to report harmful content) (20). This trend surprised us, since we had intentionally sought and included texts that made some overt mention of how online hate speech should be mitigated. In effect, despite recognizing that online hate speech is bad, our three groups of stakeholders only lightly engaged with who or how it should actually be detected or addressed. Although this lack of discussion of how identifying hate speech is actually accomplished may just be a function of the relative concerns of NGOs (i.e., advocacy and civil rights) and governments (i.e., citizen security) compared to platforms (i.e., functionality and user experience), it is startling due to the fact that accurate detection of hate speech cannot be assumed. Computer scientists have not resolved the detection problem yet, and we found it striking that these other stakeholders presumed that detection was easily accomplished or justifiably ignored.
2) While stakeholders agree that platforms are responsible for responding to hate speech, they do not agree on who decides how they respond. Both governments and platforms want to single-handedly decide how to respond to online hate speech, resulting in a power dispute between the two stakeholders. Consequently, there is no conversation among stakeholders about how to best respond to online hate speech; stakeholders are mostly working in a vacuum, rather than trying to get on the same page.

This final point underscores a key takeaway of our analysis, which is that, outside of computer science, there is virtually no discussion of automated hate speech detection—as a tool for mitigating hate speech or for any other use. This is concerning: Detection is clearly a complicated technical, ethical, and practical issue, but one that is not being addressed in key discourse.

## The Way Forward

This is where the computer science community comes in. As they have significant technical knowledge about identifying hate speech—as well as the creativity to come up with new and innovative methods that work at the scale of the hate speech problem—computer scientists have a responsibility to work with other stakeholders, like the ones we studied but also other research communities, to fully tackle the problem of online hate speech.

This does not mean abandoning detection entirely, but does involve asking other groups involved in the fight against hate speech what kind of tools they actually need or want. Crucially, other stakeholders have very real concerns about governing the response to hate speech that many computer scientists have yet to consider in their focus on automated detection. By not only engaging but facilitating dialogue with these other stakeholders, the computer science research community can play a crucial role in getting everyone on the same page when it comes to an understanding of online hate speech. In turn, other communities can engage with computer science and expand their comprehension of what tools currently exist or are being developed to detect and understand online hate speech. This dialogue would help everyone gain an informed understanding of hate speech, and pave the way to actually, finally effectively addressing it.

The alternative, i.e., continuing to work on hate speech in isolation from one another, is dangerous. As stakeholders develop incongruent understandings of hate speech and its effects, online hate speech will simply continue to proliferate and more strongly influence our online societies. Technical innovation (like that from the CS community) that does not accommodate legal frameworks or ethical concerns, and policy that does not reflect the most current technology, are equally ineffective in creating real change.

## Outw with the Methods, in with the Solutions

We therefore propose a paradigm shift to the CS community: stop thinking about online hate speech as something requiring methods, and start thinking about it as something that demands solutions. This change—treating hate speech less like a task and more like the real-world problem it is—would orient CS research towards the concerns of other stakeholders, and thus begin the collaborative pursuit toward a safe Internet.

In other words, research on online hate speech needs to happen in context: hate speech is a real problem with real impacts, and the CS community needs to orient their research to actually addressing it *as* a problem. Crucially, they cannot maintain this orientation alone. Rather, they must engage in real interdisciplinary collaboration to solve the problem of online hate speech. For example, a collaboration between computer scientists and a nonprofit could involve systematically studying (and yes, detecting) online hate speech in a specific community, allowing for the creation and propagation of effective counterspeech. Or, a collaboration between computer and political scientists may shed light on how online hate speech affects and evolves during electoral campaigns, thus advising policy. Computer scientists could even help platform designers develop robust crowd-sourced content moderation systems that use users’ understandings of their own communities to stop hate speech, work that would entirely sidestep the use of automated hate speech detection.

This collaboration is beyond fundamental. To solve online hate speech, we must understand why it's a problem and explore how computation can be part of the solution. The effectiveness of a solution for online hate speech cannot simply be measured by its accuracy or speed, but should also include an understanding of the impact of implementing that solution. CS papers on hate speech must treat the impact of their work as equally important as the work itself. For example, if a paper proposes automated detection, it should also authentically evaluate the impact of implementing that solution. Within hate speech, every method has an application—it is not sufficient or responsible to simply focus on the method.

We understand that this kind of work is already happening on a small scale, but over the past decade we have observed little progress in the spread of these practices. Change must be more deliberate and systemic across the larger computer science research community. Occasional attempts at interdisciplinary collaboration are not sufficient. Researchers must make a concentrated effort to orient their work in the real world. The CS community will not be alone in this endeavor: social science, for instance, has already heard this call to action for solutions-oriented research (21). For example, conferences focused on developing holistic/comprehensive understandings of and solutions for hate speech can bring together computer and social scientists, industry players, and government actors. We call this “end-to-end” thinking, where topics are approached by understanding the source of the problem (i.e., what features of online platforms allow for hate speech to occur?) and then broken down and pursued until a long-term solution is reached (i.e., how do we ensure safe online spaces?). It is worth noting that this type of thinking will likely slow down the rate of computational publications regarding online hate speech; we hope that, by spending more time carefully considering the many layers of this complex problem, computer scientists will produce more directly applicable and impactful work than before. Consequently, the burden of responsibility for ensuring CS research is actually useful lies not just on the researchers themselves, but on the journals and conferences who publish their work: if these venues continue to encourage work on raw hate speech detection methods with little consideration for how these methods are meaningfully situated within workable solutions, they are incentivizing researchers to distance themselves from helping to create real solutions. The CS community has the opportunity to play a significant role in developing a general understanding of the nature and impact of online hate speech, as well as shaping what solutions are proposed and implemented in the pursuit of a healthy digital ecosystem. We urge them to take it.

## Conclusion: A World Without Hate Speech

A world where hate speech is sporadic and rare is a world in which all stakeholders work together. Although our proposed solution would require a re-orientation for the CS field, away from method-based thinking and towards collaborative solutions, such a change is necessary—if the CS community stays in a bubble, its work may all be for naught, and the possibility of civil online spaces remains distant. But a world in which researchers, governments, companies, and advocacy organizations work together to overcome everyone’s concerns and challenges is a world in which we can actually do something about online hate speech and build thriving, healthy online communities where we need them. A bright future, indeed.

## Supplementary Material

Appendix 01 (PDF)

## Data Availability

All study data are included in the article and/or *SI Appendix*.
