# Supplementary material for: Is hate speech detection the solution the world wants?
Source: Proc Natl Acad Sci U S A. 2023 Feb 27;120(10):e2209384120. doi: 10.1073/pnas.2209384120 (PMC10013846; doi:10.1073/pnas.2209384120)
Supplement: Supplementary file 1 — Appendix 01 (PDF) [file pnas.2209384120.sapp.pdf]

Supporting Information for

Perspective: Is hate speech detection the solution the world wants?

Sara Parker

Derek Ruths, PhD.

Derek Ruths

Email: derek.ruths@mcgill.ca

This PDF file includes:

Appendix A

Appendix B

SI Appendix A

In this Appendix, we elaborate on our methodology for our section on the first page of the manuscript regarding the computer science's current focus on automated hate speech detection. There are many different ways of sampling a literature. We selected two methods that would both capture the community-level trends in focus and also reflect what someone would do if they were interested in finding computational work on online hate speech, which is presumably how individuals from other stakeholder communities would credibly come to an understanding of and relationship with the CS literature of this topic. Those two techniques for identifying papers are: surveying the proceedings of popular conferences and consulting prominent research databases. This resulted in manually examining (326 papers). Given our decade-long engagement in this field of research (hate speech from a CS perspective), we are confident that the trends we uncovered accurately reflect those in the computer science research community where hate speech is concerned.

Conference Proceedings

First, we considered a set of the most prominent conferences for applied ML where computer scientists might publish work on machine learning. As there is no such canonical list of conferences, we selected six conferences that spanned machine learning (NeurIPS), natural language processing (EMNLP and NAACL), and computational social science (ICWSM, WSDM, and WWW). we deemed "top six machine learning conferences" on page 2 of the main manuscript, as well as the exact numbers of papers they featured about online hate speech and how many were about detection.

For each conference, we found the list of proceedings per year and searched for papers with titles featuring "hate speech", "offensive", "toxic", or "slur." If we confirmed the paper actually concerned online hate speech (in contrast to adversarial attacks for example), we examined the paper to determine if it was exclusively focused on automated detection methods or concerned another topic related to hate speech. These are our findings.

a. NeurIPS
    i. 2021: no papers about online hate speech
    ii. 2020: 1/1 hate speech paper about detection
    iii. 2019: no papers about online hate speech
b. ICWSM
    i. 2022: 3/3 about detection
    ii. 2021: 2/2 about detection
    iii. 2020: 5/6 about detection
    iv. 2019: 0/1 about detection
c. WSDM
    i. 2022: 1 tutorial about combating hate speech
    ii. 2021: 1/1 about detection
    iii. 2020: 1/1 about detection
    iv. 2019: 1/1 about detection
d. WWW
    i. 2022: 1/2 about detection
    ii. 2021: 4/4 about detection
    iii. 2020: no papers about online hate speech.
    iv. 2019: 3/3 about detection
e. EMNLP
    i. 2021: 2/2 about detection
    ii. 2020: 3/3 about detection

iii. 2019: 1/2 about detection

f. NAACL

i. 2022: 6/6 about detection

ii. 2021: 2/2 about detection

iii. 2020: no conference held.

iv. 2019: 2/3 about detection

Computer Science Literature Databases

In our second approach, we consulted two different research databases: the AITopics research database, the official publication of the Association for the Advancement of Artificial Intelligence, and the Association for Computing Machinery's Digital Library.

For each, we considered papers published between January 1, 2019 and December 5, 2022 (to reflect the most up-to-date research). We obtained these papers using searches for three sets of keywords: "hate speech", "offensive language", and "slur". We sorted results by Relevance. After a certain number of papers, results became irrelevant to the study of online hate speech (e.g., papers beyond this point were on topics such as cybersecurity or simply happened to contain the keyword, but in a tangential way), so we stopped our count at this threshold. The threshold is reported in each keyword's results below.

AITopics. This is one of the most extensive databases of machine learning research articles, news articles, and other materials on the Internet. We filtered search results to only get research articles cross-listed in arXiv (to assure that we were examining computer science research articles, rather than news articles or statements from tech companies)..

- Hate speech: yielded 338 results. Sorted by relevance, we examined the first 50 results to determine if they were a) about online hate speech and b) not exclusively focused on hate speech detection methods. Out of the first 50 results, 6 were not exclusively focused on detection. These papers are:
    - Qian et al., "A Benchmark Dataset for Learning to Intervene in Online Hate Speech," 2019.
    - Mathew et al., "HateXplain: A Benchmark Dataset for Explainable Hate Speech Detection," 2022.
    - Akhtar, Basile, and Patti, "Whose Opinions Matter? Perspective-aware Models to Identify Opinions of Hate Speech Victims in Abusive Language Detection," 2021.
    - Chung et al., "Empowering NGOs in Countering Online Hate Messages," 2021.
    - Han et al., "American Hate Crime Trends Prediction with Event Extraction," 2021.
    - Kiritchenko, Nejadgholi, and Fraser, "Confronting Abusive Language Online: A Survey from the Ethical and Human Rights Perspective," 2020.
- Offensive language: yielded 427 results. Papers began to become irrelevant to online offensive language after the first 30 results, so we only examined the first 30 papers. Of these 30 papers, 3 were not exclusively focused on detection methods. These papers are:
    - Excell and Moubayed, "Towards Equal Gender Representation in the Annotations of Toxic Language Detection," 2021.
    - Kiritchenko, Nejadgholi, and Fraser, "Confronting Abusive Language Online: A Survey from the Ethical and Human Rights Perspective," 2020.
    - Mirsky et al., "The Threat of Offensive AI to Organizations," 2021.
- Slur: yielded 19 results. Only 13 considered natural language processing, while 7 were related to online hate speech. Of these 7 papers, only 3 were not exclusively focused on detection methods. These papers are:

- Mishra, Yannakoudakis, and Shutova, “Modeling Users and Online Communities for Abuse Detection: A Position on Ethics and Explainability,” 2021.
- Kou and Gui, “Mediating Community-AI Interaction through Situated Explanation: The Case of AI-Led Moderation,” 2020.
- Weidinger et al., “Ethical and social risks of harm from language models,” 2021.

ACM Digital Library. We then consulted the Association for Computing Machinery’s Digital Library, using the same keywords and search parameters. The ACM DL is larger than the AAAI database, primarily because it includes papers outside of the computer science discipline. We again filtered by Relevance because we received search results that were not relevant to the study of online hate speech through any other method of sorting.

- Hate speech: yielded 13,496 results. We examined the first 60 papers (three pages of search results), after which results began to be irrelevant. Of these 60 papers, 12 were not exclusively focused on detection methods. Of these 12 papers, 7 were computer science papers - i.e., written by one or more computer scientists. These papers are:
    - Das et al., “Hate speech in online social media,” 2020.
    - Mathew et al., “Spread of Hate Speech in Online Social Media,” 2019.
    - Masud et al., “Proactively Reducing the Hate Intensity of Online Posts via Hate Speech Normalization,” 2022.
    - Agarwal et al., “Hate Speech in Political Discourse: A Case Study of UK MPs on Twitter,” 2021.
    - Mathew et al., “Interaction dynamics between hate and counter users on Twitter,” 2020.
    - Das et al., “You too Brutus! Trapping Hateful Users in Social Media: Challenges, Solutions, and Insights,” 2021.
    - Petrescu et al., “Sparse Shield: Social Network Immunization vs. Harmful Speech,” 2021.
- Offensive language: yielded 42,876 results. We examined the first 60 papers, after which results became irrelevant to online offensive language. Of these 60 papers, 4 were not exclusively focused on detection methods. Of these 4, 3 were computer science papers. These papers are:
    - Balayn et al., “Automatic Identification of Harmful, Aggressive, Abusive, and Offensive Language on the Web: A Survey of Technical Biases Informed by Psychology Literature,” 2021.
    - Weidinger et al., “Taxonomy of Risks posed by Language Models,” 2022.
    - Alshamrani et al., “Hate, Obscenity, and Insults: Measuring the Exposure of Children to Inappropriate Comments in YouTube,” 2021.
- Slurs: yielded 165 results. We examined the first 60 papers, after which papers became irrelevant to online slur usage. Of these 60 papers, 17 papers regarding slur usage online were not exclusively focused on detection methods. Of these 17, 13 were computer science papers. These computer science papers are:
    - Mathew et al., “Spread of Hate Speech in Online Social Media,” 2019.
    - Jhaver et al., “Designing Word Filter Tools for Creator-led Comment Moderation,” 2022.
    - Kou and Gui, “Flag and Flaggability in Automated Moderation: The Case of Reporting Toxic Behavior in an Online Game Community,” 2021.
    - Harris et al., “Exploring the Role of Grammar and Word Choice in Bias Toward African American English (AAE) in Hate Speech Classification,” 2022.
    - Chandrasekharan et al., “Quarantined! Examining the Effects of a Community-Wide Moderation Intervention on Reddit,” 2022.

- Balayn et al., “Automatic Identification of Harmful, Aggressive, Abusive, and Offensive Language on the Web: A Survey of Technical Biases Informed by Psychology Literature,” 2021.
- Das et al., “You too Brutus! Trapping Hateful Users in Social Media: Challenges, Solutions, and Insights,” 2021.
- Panda et al., “Affording Extremes: Incivility, Social Media and Democracy in the Indian Context,” 2020.
- Juneja, Subramanian, and Mitra, “Through the Looking Glass: Study of Transparency in Reddit’s Moderation Practices,” 2020.
- Kou, “Punishment and Its Discontents: An Analysis of Permanent Ban in an Online Game Community,” 2021.
- Masud et al., “Proactively Reducing the Intensity of Online Posts via Hate Speech Normalization,” 2022.
- Alkhatib and Bernstein, “Street-Level Algorithms: A Theory at the Gaps Between Policy and Decisions,” 2019.
- Ribeiro et al., “Do Platform Migrations Compromise Content Moderation? Evidence from r/The_Donald and r/Incels,” 2021.

We found it interesting that computer science research on slurs was overall less concerned with detection methods than research on offensive language or hate speech, even though slurs are a category of hate speech. We believe that this may be because the study of slurs requires some understanding of community factors – in essence, one must grasp the sociological components of the usage of a slur to even know where to look for it. As a result, computational research *specifically on slurs* demonstrates an interdisciplinary effort to understand the phenomenon, rather than simply develop automated detection methods. Computational research on slurs therefore potentially represents a latent community of computer scientists interested in solution-oriented work and an important first step to solution-oriented computer science.

The papers mentioned in this section show two things. First, automated hate speech detection is by far the prevailing research trend among computer scientists working in this area. Second, there are some computer scientists interested in studying online hate speech, beyond the development of automated detection methods. However, we must reiterate the importance of a community-wide shift in approach to online hate speech towards solutions. Although many of these researchers are doing important work to broaden computational research on online hate speech, we argue that the community as a whole could be doing more to directly engage the problem as it exists in the real world.

SI Appendix B

In this Appendix, we provide the full details for how our discourse analysis was conducted - from how textual sources were identified and collected to the results (highlighted in the main manuscript) obtained as a result of the analysis.

To briefly revisit, the objective of our discourse analysis was to deconstruct and thereby characterize the ways that different stakeholder communities discuss the problem of online hate speech and how to address it. This process is sometimes called "discourse mapping" and can take many different forms depending on the goal of the research, the data available, and the nature of the discourse. With this in mind, our method most closely resembles that of Kuhn (2020), Certoma and Corsini (2021), and Redden (2018). Kuhn (2020) evaluates texts from governments, financial institutions, and NGOs and identifies the key themes that connect the stakeholders regarding sustainable finance in Germany. Like Kuhn, our key stakeholders of interest were governments, NGOs, and the private sector actors most relevant to our topic of study (i.e., social media platforms:hate speech). Certoma and Carsini (2021) use a Foucaudian method of discourse analysis, emphasizing the relations of power and knowledge across various actors, to map an "emerging discursive field" about digital social innovation. The Foucaudian approach to discourse, with a focus on social power relations, was particularly important to us throughout the project: as solutions for online hate speech must be decided and implemented by somebody - whether a platform, government, or individuals themselves - we believed that it was important to identify who *could* make these decisions and who actually *was* making them. Consequently, we kept Foucault in mind when designing our project, and thus carefully considered how stakeholders were asserting their power (or lack thereof) through discourses. Finally, Redden (2018) engages in a qualitative research case study to map Big Data practices and internal discussions of the Government of Canada. Redden's focus on understanding how government actors thought about data governance partially mirrors our approach to understanding how stakeholders think about online hate speech, although our work focuses on a much larger discursive community than just the Government of Canada.

The rest of this Appendix explains the phases of the discourse mapping project. We provide this information in the case that other researchers may want to replicate our method to conduct their own discourse mapping.

The phases of the project were as follows:

1. Stakeholder groups identification
2. Text selection
3. Creating the sample
4. Discourse analysis
5. The Character-Role framework
6. Coding/annotating texts (the Matrix)
7. Results/discourse mapping

<u>1. Stakeholder groups identification</u>

First, we identified our key stakeholders of interest. We wanted to see if we could identify thematic trends within content produced by these stakeholder groups ("discursive communities") and therefore chose to study them separately.

- Governments: as a discursive community, this refers to any organization or member of an organization that serves as a governing body. We therefore have a range of sources designated as "Government", from candidates for the Democratic nomination for the President of the United States to official reports from the United Nations. We lumped them together because these sources occupy similar space in social power relations as capable of driving legislation and regulation that affects individuals and platforms.

- Platforms: as a discursive community, this refers to social media platforms where one may find instances of online hate speech. Although some platforms have more hate speech and more users than others, they all have the unilateral power to enforce content moderation policy on their sites.
- Non-governmental organizations (NGOs): as a discursive community, this refers to a variety of non-governmental organizations that have done advocacy work related to online hate speech. We included NGOs that advocate for free speech (and would therefore be less likely to support the removal of hate speech) and those that advocated for minority groups (and would therefore be more likely to support the removal of hate speech directed towards a particular group).

We did not include other stakeholder groups, such as commercial clients of platforms, due to a lack of publicly accessible content regarding their stance on online hate speech. Additionally, although we discussed focusing our stakeholder groups to a specific country or region (e.g., Canada or North America), we decided against this so as to give ourselves the freedom to collect as many samples as possible.

<u>2. Text selection:</u>

Once we determined what kind of discourse we wanted, we began to search for text samples from each discursive community. The texts were collected from manual online keyword searches using Google. The first set of samples featured a preliminary collection of results explicitly featuring the words “hate speech detection”; we soon realized that many texts implicitly referred to hate speech detection, allowing us to broaden our search to texts featuring hateful content more generally. The links to the texts were then saved in a file, with brief notes explaining the source (e.g., “Government - Canada”) and the subject of the text (e.g., “Introducing legislation about online hate speech”).

It is worth noting that finding texts that pertained to addressing online hate speech in any way was exceedingly difficult and labor-intensive given the high volume of content about online hate speech in general. Many sources simply asserted online hate speech as existing or being a problem, but did not actually mention anything about how they were or planned to address it. Such texts that did not discuss a response to online hate speech were not included in our initial sample.

<u>3. Creating the sample</u>

Our initial sample of texts included over 50 texts, which we divided into categories based on the stakeholder source. We then conducted a first reading of the texts to ensure that they were actually about addressing online hate speech, particularly if they (even implicitly) included the question of hate speech detection. Texts that did not discuss addressing hate speech were removed from the sample as the goal of the project was to understand how stakeholders thought about hate speech solutions, not just hate speech in general.

This final sample featured 35 unique texts that discuss, reference, or feature hate speech response as a key theme. 10 are from government sources, and range from official national legislation to party platforms to government-commissioned reports. 13 are from non-governmental organizations (NGOs) that advocate for free speech, privacy, and/or minority rights. These texts address both the general phenomenon of hate speech and specific contexts in which hate speech occurs, while providing recommendations or calls to action. Finally, 12 texts are from social networking platforms, including official policies on hateful content and releases/articles/blog posts from the platform about hate speech detection. Citations for all included texts can be found in the final section of this Appendix.

<u>4. Discourse analysis</u>

All texts in the final sample were then carefully read multiple times. The reader took detailed notes summarizing the texts and identifying what stakeholders were discussed and what they were doing in the text. For example, if a text from an NGO condemned a law for violating freedom of expression, the notes looked like this:

- NGO condemning new law in (country) that prohibits online hate speech on grounds of freedom of expression violation
- Government - violating freedom of expression
- Individuals - rights violated

This method of analysis allowed us to identify common themes that appeared in the texts, leading to the creation of the Framework in the following section.

5. The Character-Role framework

We then put together all the stakeholder-action notes into one document and read them to find actions that overlapped (e.g., Governments and Platforms both violating freedom of expression). We then put them into five thematic categories called “roles”, which we derived from our interpretation of the notes: Villains (perpetrating a harm), Victims (subject to harm), Responder (responding to online hate speech), Detector (finding online hate speech), and Governor (overseeing responses to online hate speech). These roles were then broken down into sub-categories to provide details about what exactly made a stakeholder (a “character”) play a given “role.”

The complete role breakdown is as follows:

Villain: There are three types of Villains.

- Villain 1 hosts and/or disseminates hate speech and is always “played” by a platform. This means that, in the texts featuring Villain 1, platforms are treated as principally responsible for the spread of hate speech and must therefore be responsible for detecting and mitigating it.
- Villain 2 perpetrates and/or engages in hate speech and is always an individual. In discourses featuring Villain 2, individuals are held responsible for the existence and spread of hate speech. In essence, the discourse emphasizes individuals producing hateful content as the main cause of hate speech. This is the most common type of Villain.
- Villain 3 is not responsible for hate speech, but rather for the violation of freedoms, and can be “played” by a government or platform. These discourses often critique a specific policy proposed or implemented by a government or platform on the grounds that it poses a real or potential threat to freedom of expression and/or privacy.

Victim: The victim is always an individual and can fall into two categories.

- Victim 1 is the victim of hateful content. In discourses featuring Victim 1, the main subject of concern is the effect of hate speech on targeted individuals. This is the most common type of Victim.
- Victim 2 is subject to a rights violation. In these texts, individuals are portrayed as having their freedom of expression and/or privacy violated by a government or platform. Discourses with Victim 2 often also feature Villain 3.

Detector: there are three types of detector, and are either individuals or platforms. Notably, although all texts in our sample discuss hate speech detection - explicitly or implicitly - not all texts assign a Detector role.

- Detector 1 is always an individual and reports instances of hate speech to the platform hosting the content. In these texts, individual users bear the burden of responsibility for detecting hate speech; hate speech detection is therefore manual and regulated primarily by the users of the platform, rather than automated and *determined* by the platform itself. This is the most common type of Detector.
- Detector 2 is a platform who automatically detects hate speech, often without requiring (or mentioning the need for) complaints from users. These discourses primarily originate from the platform itself, indicating that some platforms appear to voluntarily undertake the responsibility of hate speech detection. This type of detector is closest to what a computer scientist may consider a practical application of automated hate speech detection.
- Detector 3 is a "catch-all" category, referring to those discourses that feature a vague concept of content moderation attributed to platforms or individuals. In contrast to the other two types of Detectors, the assignment of this role does not entail a specific action, but rather assigns responsibility for fostering and maintaining a peaceful digital environment through content moderation (i.e., removal of and response to hate speech).

Responder: There are four types of responder.

- Responder 1 responds to hate speech by monitoring or working to understand it. This role is played by platforms and/or NGOs (sometimes working together) and is often portrayed as a careful first step to addressing the problem of hate speech. These discourses often do not assign responsibility to NGOs or governments for understanding, but rather describe a potential or active role in studying hate speech.
- Responder 2 directly responds to hate speech by punishing the perpetrator and/or removing the content directly; it is therefore always a platform. This is the most common type of Responder, suggesting that, in the majority of discourses in our sample, platforms are thought to be most responsible for addressing the problem of hate speech.
- Responder 3 engages in counterspeech or education initiatives, and can be played by a NGO, individual, or the government. In contrast to the other types of Responders, this role is less concerned with hateful content *itself* and more concerned with the content that mitigates its effects.
- Responder 4 reports information on hate speech to the government or the public and is therefore always a platform. In the texts in our sample that feature this role, Responder 4 is held to a legal obligation to report hateful content on their platform and how they responded to it.

Governor: the most nuanced role, there are five types of governor.

- Governor 1 creates and/or enforces a rule relating to hate speech, and can be either a government or a platform. When attributed to a government, this role mostly refers to the creator and enforcer of a law governing hate speech, either through the prohibition of hateful content online (individuals are subject to the law) or obligating platforms to detect, respond to, and report hate speech on their sites (platforms are subject to the law). This is the most common type of Governor.
- Governor 2 receives reports from platforms about hate speech detection, and is therefore always a government. These reports are intended to provide transparency to the government - and the public - about how platforms address and regulate hate speech on their platforms, and allow the government to hold platforms accountable for their approaches to hate speech detection.
- Governor 3 works to understand hate speech, but in a distinct way from Responder 1. In contrast to Responder 1, discourses featuring Governor 3 attribute a burden of responsibility to governments to understand what drives hate speech and how it impacts their citizens.

- Governor 4 emphasizes collaboration with other stakeholders, and can thus refer to platforms, individuals, and/or government (and presumably NGOs). These texts often discuss the importance of platforms collaborating with individuals to address hate speech, suggesting a hybrid automated-manual approach to hate speech detection.
- Governor 5 asserts the role of platforms in governing their site. Discourses featuring Governor 5 shy away from assigning specific responsibility for action addressing hate speech, but rather assert the principle of platform self-governance.

6. Coding/annotating texts (the Matrix)
The sub-categorized roles played by the characters/stakeholders were then identified in each text based on the detailed notes written in Phase 4. For each text, we identified which character/stakeholder appeared, what role that character played, and then what sub-role they played. This coding was represented in a detailed table, which we call the Matrix and can be seen below in Figure 1. Six months later, each text was read again by the same reader/coder, who then identified characters and assigned roles (skipping the note-taking stages), creating another table and ensuring the validity of the initial results.

Figure 1: The Matrix. This table contains the application of the Character-Role framework to each text in our final sample. The numbers in parentheses next to each Character indicates the sub-category of role they play in the text, as explained in the previous section. Some texts have multiple rows because they assign the same role to multiple characters.

	**ROLE**					**STAKEHOLDER**
**TEXT**	Villain	Victim	Detector	Responder	Governor	
Canada's Digital Charter	Platform (1)	Individual (1)				Government
UNESCO: Countering online hate speech				Government (1)	Government (4)	Government
UNESCO: Countering online hate speech				NGO (3)	NGO (4)	
UNESCO: Countering online hate speech				Platform (1)		
UNESCO: Countering online hate speech				Individual (3)		
Germany's Network Enforcement Act (NetzDG)			Individual (1)	Platform (2)	Government (1)	Government
Germany's Network Enforcement Act (NetzDG)				Platform (4)	Government (2)	
Liberal Party of Canada: Protecting Canadians from Online Harms	Platform (1)	Individual (1)			Government (1)	Government
European Commission: Code of conduct on			Individual (1)	Platform (2)	Government (1)	Government

countering hate speech online						
European Commission: Code of conduct on countering hate speech online				NGO (3)	NGO (4)	
European Commission: Code of conduct on countering hate speech online					Government (4)	
USCIRF: Combatting Online Hate Speech and Disinformation Targeting Religious Communities	Platform (1)	Individual (1)			Government (1)	Government
OHCHR: Report on online hate speech	Government (3)			Platform (2)	Government (1)	Government
Department of Justice Canada: takes action to protect Canadians		Individual (1)		Platform (2)	Government (1)	Government
2020 Democrats: responsibility for misinformation and hate speech	Platform (1)				Government (1)	Government
2020 Democrats: responsibility for misinformation and hate speech					Government (4)	
UN: Strategy and Plan of Action on Hate Speech		Individual (1)		Government (1)	Government (4)	Government
HRW: Germany Flawed Social Media Law	Government (3)	Individual (2)				NGO
Amnesty: Facebook and Google surveillance	Platform (3)	Individual (2)				NGO
Amnesty: Ethiopia and Tigray conflict	Government (3)	Individual (2)				NGO
Amnesty: Ethiopia and Tigray conflict	Government (2)	Individual (1)				NGO
Amnesty: Vietnam cybersecurity law	Government (3)	Individual (2)				NGO
Amnesty: Facebook					Platform (5)	NGO

must help lead fight						
Amnesty: Silence Hate Poland				Individual (3)		NGO
Amnesty: Silence Hate Poland				NGO (3)		
Santa Clara Principles	Individual (2)			Platform (2)		NGO
Santa Clara Principles				Platform (4)		
EFF: Indonesia's Proposed Online Intermediary Regulation	Government (3)	Individual (2)				NGO
Stop Hate UK: How to Challenge Online Hate	Individual (2)	Individual (1)		Individual (3)		NGO
ACLU: Content Moderation	Platform (3)	Individual (2)				NGO
Anti-Defamation League: Online Hate and Harassment	Platform (1)	Individual (1)				NGO
NAACP: Stop Hate for Profit and Facebook	Platform (1)	Individual (1)		Platform (2)		NGO
				Platform (1)		
CivilRights.org: Combat Hate Speech	Platform (1)			Individual (3)		NGO
Facebook: super-efficient AI models to detect hate speech			Platform (2)			Platform
Facebook: Community Standards, Hate Speech Policy	Individual (2)	Individual (1)		Platform (2)	Platform (1)	Platform
Twitter: HateLab			Platform (2)	NGO (3)	Platform (4)	Platform
Twitter: Rules	Individual (2)	Individual (1)	Individual (1)	Platform (2)	Platform (1)	Platform
Reddit: understanding hate on Reddit	Individual (2)		Platform (2)	Platform (2)	Platform (1)	Platform
					Platform (3)	
TikTok: Community Guidelines	Individual (2)			Platform (2)	Platform (1)	Platform

Pinterest: How we fight misinformation, hate speech, and self-harm content with ML			Platform (2)	Platform (2)	Platform (1)	Platform
Parler: Community Guidelines					Platform (5)	Platform
WhatsApp: How to stay safe on WhatsApp			Individual (1)	Platform (2)	Platform (1)	Platform
Tumblr: Community Guidelines	Individual (2)		Individual (1)	Platform (2)	Platform (1)	Platform
Xbox: Community Standards	Individual (2)			Platform (2)	Platform (1)	Platform
					Platform (4)	
LinkedIn: Professional community policies	Individual (2)			Platform (2)	Platform (1)	Platform
YouTube: Hate speech policy	Individual (2)			Platform (2)	Platform (1)	Platform

7. Results/discourse mapping

Once the Matrix was created, we could quickly identify trends in character-role assignments within discursive communities, giving us a detailed idea of how these stakeholders thought about addressing hate speech. We tallied how many times each role appeared in the texts from each community (e.g., 70% of texts from Governments feature a Responder) and used these numbers to identify trends in the discourses.

Finally, we spent a considerable amount of time reviewing and consulting the Matrix, identifying patterns we saw and discussing their potential implications. We cannot overstate the importance of visualizing the discourses in identifying the discursive trends discussed in the section “Making sense of the mess”. Being able to easily see what stakeholders thought to be the key issues regarding online hate speech (e.g., an emphasis on harm conducted against individuals vs. an emphasis on response) gave us insight that we would not have had without the Matrix. This systematic approach to reading and understanding texts allowed us to identify trends within communities, as well as the tensions between them; in particular, we were able to pinpoint sites of agreement (i.e., what stakeholders agreed on) and key issues on which they diverged (e.g., who is responsible for making rules regarding online hate speech).

Bibliography of the 35 sources (in alphabetical order)

1. ACLU, Time and Again, Social Media Giants Get Content Moderation Wrong: Silencing Speech about Al-Aqsa Mosque is Just the Latest Example | News & Commentary. American Civil Liberties Union (2021)
2. Amnesty International, Viet Nam: Proposed cybersecurity law threatens to stamp out online freedom. Amnesty International (2018)
3. Amnesty International, Silence Hate: a project against hate speech in Poland. Amnesty International (2019)

4. Amnesty International, Facebook and Google's pervasive surveillance of billions of people is a systemic threat to human rights. Amnesty International (2019)
5. Amnesty International, Ethiopia: Sweeping emergency powers and alarming rise in online hate speech as Tigray conflict escalates. Amnesty International (2021)
6. Anti-Defamation League, Online Hate and Harassment: The American Experience 2021
7. D. of J. Canada, Government of Canada takes action to protect Canadians against hate speech and hate crimes (2021)
8. G. of Canada, Canada's Digital Charter: Trust in a digital world (2022)
9. CivilRights.Org, Online Hate Speech is Pervasive. Here's How to Start Combating it. The Leadership Conference Education Fund
10. P. Engineering, How Pinterest fights misinformation, hate speech, and self-harm content with machine learning. Pinterest Engineering Blog (2021)
11. European Commission, Code of Conduct on Countering Illegal Hate Speech Online (2019).
12. Facebook, How Facebook uses super-efficient AI models to detect hate speech
13. I. Gagliardone, G. Danit, T. Alves, G. Martinez, Countering online hate speech - UNESCO Digital Library (United Nations Educational, Scientific, and Cultural Organization, 2015)
14. Human Rights Watch, Germany: Flawed Social Media Law. Human Rights Watch (2018)
15. Liberal Party of Canada, Protecting Canadians from Online Harms | Liberal Party of Canada
16. LinkedIn, LinkedIn Professional Community Policies
17. Meta, Hate Speech | Transparency Center
18. R. Molla, Should social media companies be legally responsible for misinformation and hate speech? 2020 Democrats weigh in. Vox (2019)
19. NAACP, Statement from Stop Hate For Profit on Meeting with Facebook | NAACP (2020)
20. OHCHR, "Report on online hate speech" (UN OHCHR, 2019)
21. Parler, Community Guidelines (2021).
22. K. Rodriguez, Indonesia's Proposed Online Intermediary Regulation May be the Most Repressive Yet. Electronic Frontier Foundation (2021)
23. Stop Hate UK, How to Challenge Online Hate. Stop Hate UK
24. TikTok, Community Guidelines
25. Tumblr, Community Guidelines
26. Twitter, HateLab
27. Twitter, The Twitter rules: safety, privacy, authenticity, and more
28. United Nations, United Nations Office on Genocide Prevention and the Responsibility to Protect
29. USCIRF, USCIRF Hearing on Combatting Online Hate Speech and Disinformation Targeting Religious Communities | USCIRF (2022)
30. WhatsApp, How to stay safe on WhatsApp | WhatsApp Help Center
31. worstnerd, Understanding hate on Reddit, and the impact of our new policy. r/redditsecurity (2020)
32. Xbox, Xbox Community Standards | Xbox. Xbox.com
33. YouTube, Hate speech policy - YouTube Help
34. Network Enforcement Act (Netzdurchsetzunggesetz, NetzDG) (2017)
35. Santa Clara Principles on Transparency and Accountability in Content Moderation. Santa Clara Principles

<u>SI Appendix B References</u>

1. Kuhn, B. M. "Sustainable finance in Germany: Mapping discourses, stakeholders, and policy initiatives." *Journal of Sustainable Finance & Investment,* 1-28 (2020).

2. Certoma, C., & Corsini, F. “Digitally-enabled social innovation: Mapping discourses on an emergent social technology.” *Innovation: The European Journal of Social Science Research* 34(4), 560-584 (2021).
3. Redden, J. “Democratic governance in an age of datafication: Lessons from mapping government discourses and practices.” *Big Data & Society* 5(2) (2018).
